# Supplementary material for: Lactational delivery of Triclosan promotes non-alcoholic fatty liver disease in newborn mice
Source: Nat Commun. 2022 Jul 27;13:4346. doi: 10.1038/s41467-022-31947-4 (PMC9329322; doi:10.1038/s41467-022-31947-4)
Supplement: Supplementary file 1 — Supplementary Information [file 41467_2022_31947_MOESM1_ESM.pdf]

# SUPPLEMENTARY DATA

## Lactational delivery of Triclosan promotes non-alcoholic fatty liver disease in newborn mice

André A. Weber[1], Xiaojing Yang[1], Elvira Mennillo[1], Jeffrey Ding[2], Jeramie D. Watrous[2], Mohit Jain[2], Shujuan Chen[1], Michael Karin[3], Robert H. Tukey[1,*].

Short Title: *Triclosan drives neonatal NAFLD*

[1]Laboratory of Environmental Toxicology, Department of Pharmacology, University of California, San Diego, La Jolla, CA 92093, USA.

[2]Departments of Medicine and Pharmacology, University of California, San Diego, La Jolla, CA 92093, USA.

[3]Laboratory of Gene Regulation and Signal Transduction, Department of Pharmacology, University of California, San Diego, La Jolla, CA 92093.

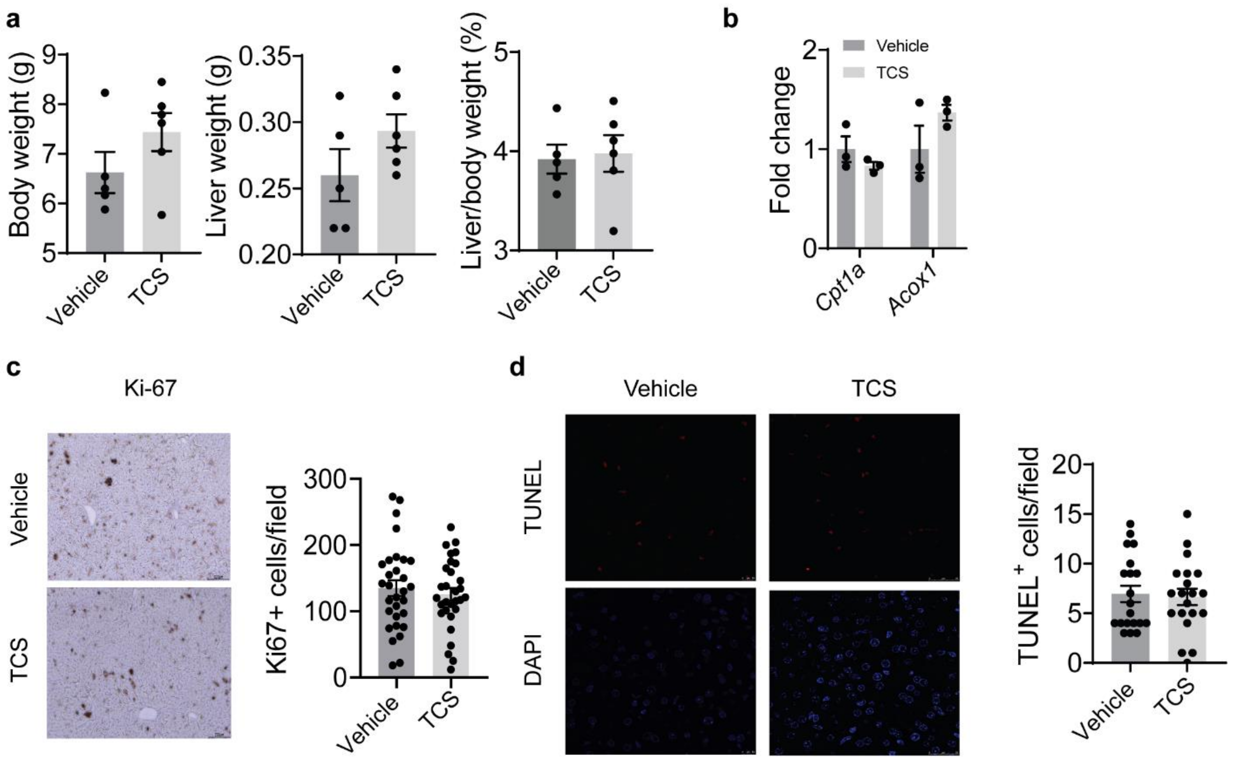


**Figure S1**. **TCS has no effect on body and liver weight and hepatic proliferation and cell death.** WT mice breastfed with vehicle or TCS for 21-days-old. **a**, Body weight, liver weight and Liver/Body Weight ratio (%) (n=5-6 mice per group). **b.** Expression of genes associated with fatty acid oxidation *Cpt1a* and *Acox1* (n=3 mice per group). **c**, Hepatocyte's proliferation was determined by Ki-67 immunostaining and quantitation of positive cells (n=30 images/5 mice per group). **d**, Liver cell apoptosis was determined by TUNEL staining and quantitation of positive cells (n=20 images/5 mice per group). Scale bars: **c**, 200µm and **d**, 20µm. **a-d** show mean ± S.E., determined by two-tailed Student`s test.

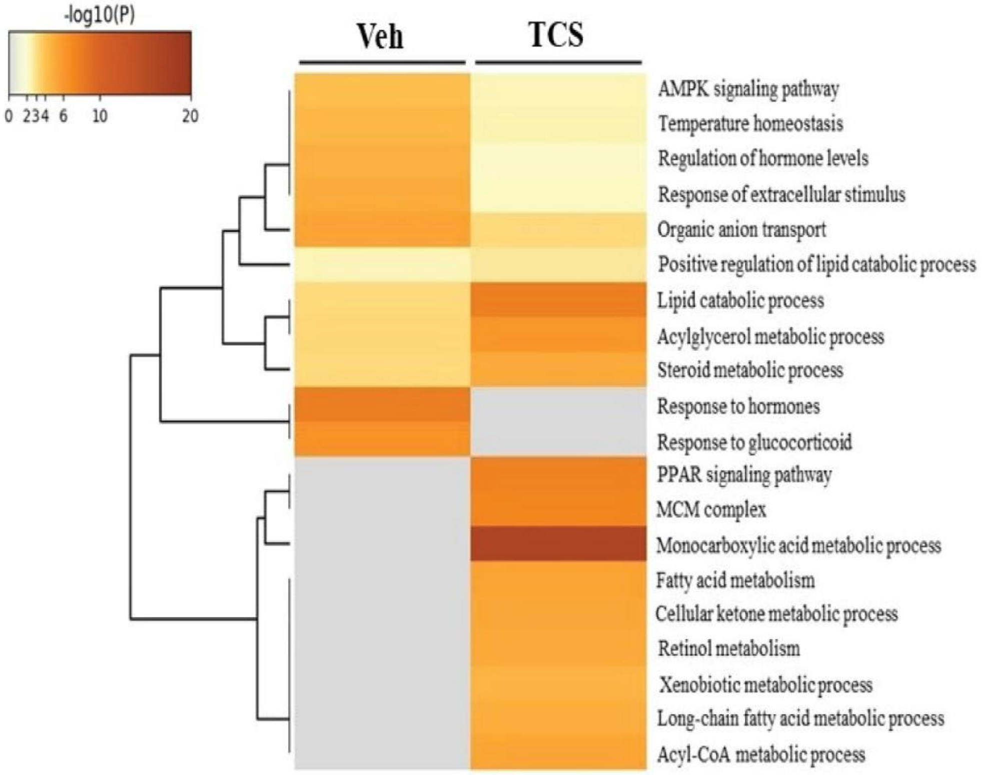


**Figure S2. TCS-induced alterations in the liver transcriptome**. WT mice breastfed with vehicle or TCS for 21-days-old. Heatmap depicting expression of HCC-related genes in liver tissue of neonatal mice treated with vehicle or TCS (n=3 mice per group). All genes showed in this figure had a *p*-adjusted value above 0.05, showing no differences between groups.

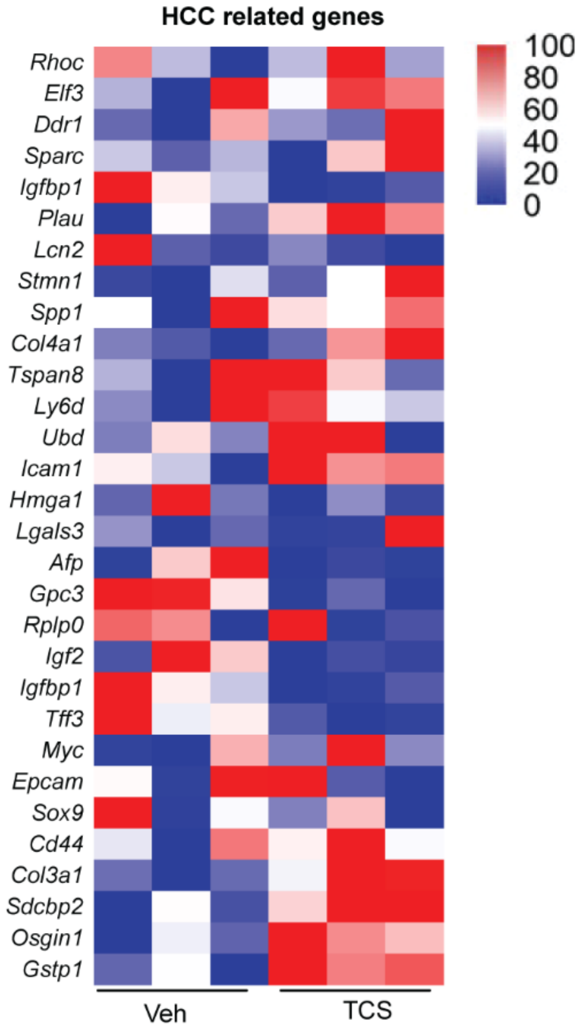


**Figure S3. TCS has little effect on HCC-related genes expression**. WT mice breastfed with vehicle or TCS for 21-days-old. Heatmap depicting expression of HCC-related genes in liver tissue of neonatal mice treated with vehicle or TCS (n=3 mice per group). All genes showed in this figure had a *p*-adjusted value above 0.05, showing no differences between groups.

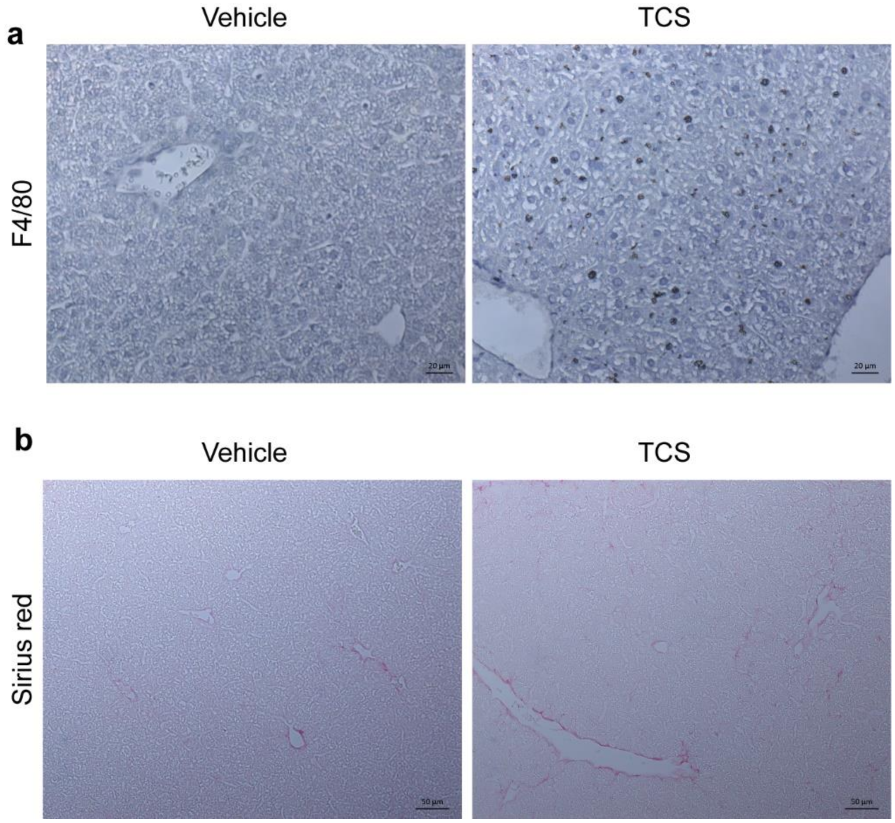


**Figure S4. TCS increases immune infiltration and fibrosis**. WT mice breastfed with vehicle or TCS milk for 21-days-old. **a**, IHC of formalin-fixed paraffin-embedded liver sections from vehicle and TCS group F4/80 immunostaining show macrophage infiltration (n=4 mice per group). **b**, Frozen sections were stained by Sirius Red to visualize collagen deposition (n=4 mice per group). Scale bars: **a**, 20µm and **b**, 50µm.

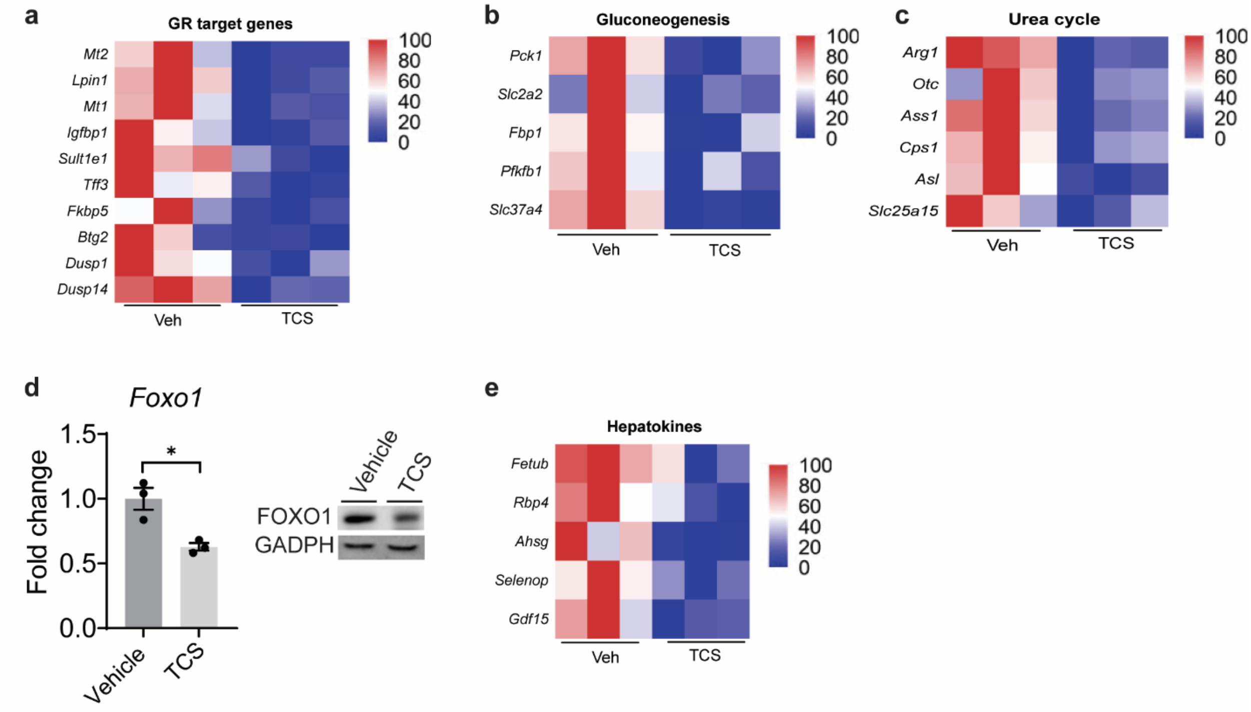


**Figure S5. TCS downregulates glucocorticoid receptor and downstream pathways.** WT mice breastfed with vehicle or TCS for 21-days-old. **a-c** Heatmaps showing differential expression of genes associated with glucocorticoid receptor (GR) signaling (**a**), and genes associated with downstream GR pathways, gluconeogenesis (**b**) and urea cycle (**c**) (n=3 per group). **d**, gene expression and representative IB image of FOXO1 in liver. **e**, Heatmap showing differential expression of hepatokines in liver (n=3 per group). **d** show mean ± S.E., determined by two-tailed Student`s test; **P*<0.05. All genes used are significant and p adjusted value below the cut-off level of 0.05.

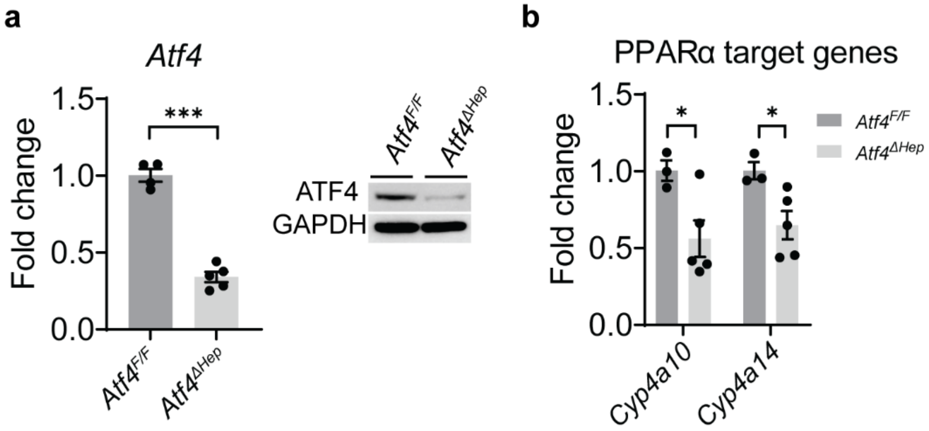


**Figure S6.** Downstream **PPARα target genes are downregulated in *Atf4*$^{\Delta Hep}$ mice treated with TCS.** *Atf4*$^{F/F}$ and *Atf4*$^{\Delta Hep}$ mice breastfed with TCS milk 21-days-old. **a**, Hepatic expression of ATF4 gene in both *Atf4*$^{F/F}$ and *Atf4*$^{\blacktriangle Hep}$ livers and IB of ATF4 (n=4-5 mice per group). **b**, Hepatic expression of downstream PPARα target genes in *Atf4*$^{F/F}$ and *Atf4*$^{\blacktriangle Hep}$ (n=3-5 mice per group). **a, b** show mean ± S.E., determined by two-tailed Student`s test; *P<0.05, ***P<0.001.

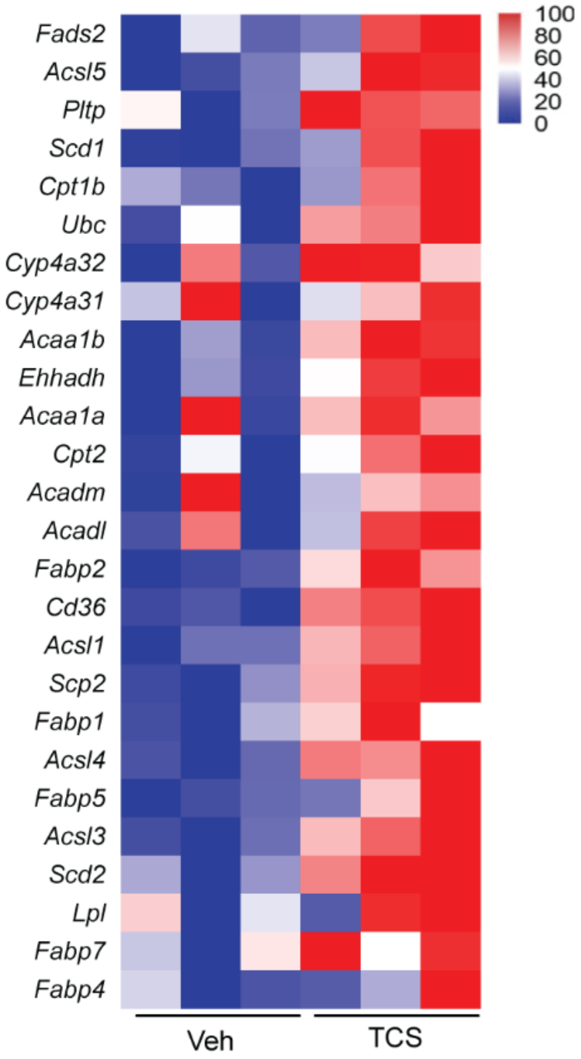


**Figure S7. TCS has strong effect on PPARα target genes.** WT mice breastfed with vehicle or TCS for 21-days-old. Heatmap depicting expression of downstream PPARα target genes in liver tissue of neonatal mice treated with vehicle or TCS (n=3 mice per group). All genes showed in this figure had a p-adjusted value above 0.05, showing no differences between groups.

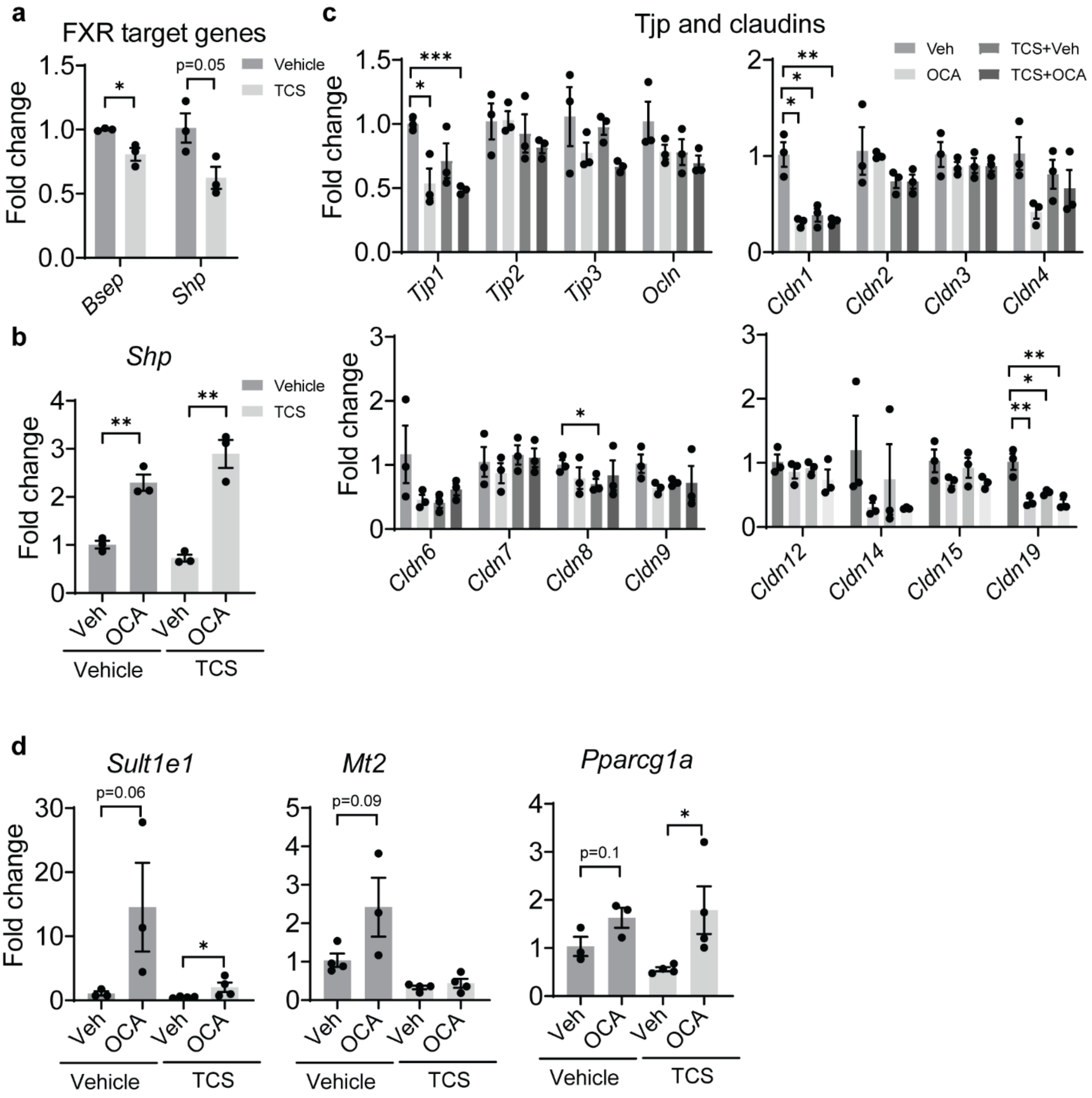


**Figure S8. OCA has no effect in intestinal barrier genes but increases GR response in liver in the absence of TCS**. WT neonatal mice breastfed with vehicle or TCS milk for 21 days and treated with vehicle or OCA. **a**, Hepatic expression of genes associated with FXR (n=3 mice per group). **b**, Intestinal expression of *Shp* (n=3 mice per group). **c**, Intestinal expression of TJPs, occludin and claudins (n=3 mice per group). **d**, Hepatic expression GR related genes (n=3-4 mice per group). **a-d** show mean ± S.E., determined by two-tailed Student`s test; *P<0.05, **P<0.01, ***P<0.001.

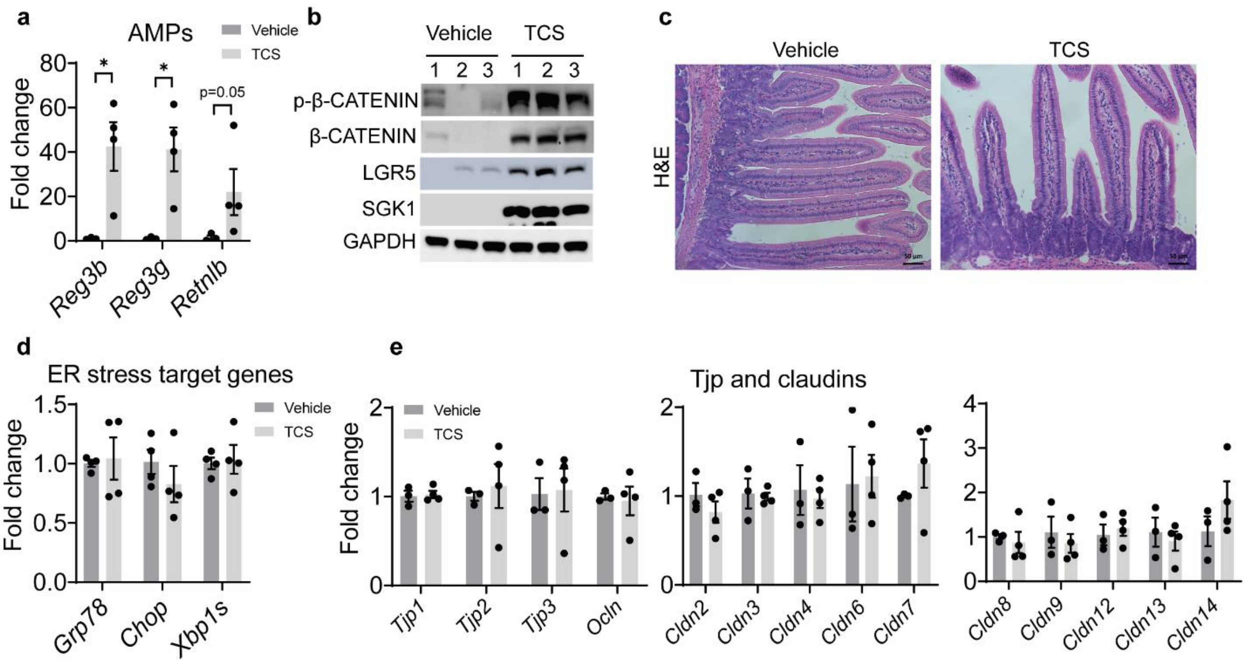


**Figure S9. TCS treatment upregulates AMPs and β-catenin pathway in intestines**. WT neonatal mice breastfed with vehicle or TCS milk for 21 days. **a**, Intestinal expression of *Reg3b*, *Reg3g* and *Retnlb* (n=3-4 mice per group). **b**, Intestinal IB of total and phosphorylated β-catenin and LGR5 and SGK1 (n=3 mice per group). **c**, Jejunum histological sections were stained with H&E (n=3 per group), Scale bars=50μm. **d,** Intestinal expression of genes associated with ER stress (n=4 mice per group). **e**. Intestinal TJP and claudins genes (n=3 mice per group). **a**, **d** and **e** show mean ± S.E., determined by two-tailed Student`s test; *P<0.05.

## Table

**Table S1. NAFLD semi-quantitative score in neonatal mice breastfed with TCS, according to Brunt (**Brunt, E.M. Nonalcoholic steatohepatitis: pathologic features and differential diagnosis. *Semin Diagn Pathol* **22**, 330-338, 2005)

| **Histopathology** | **Vehicle** | **TCS** |
|---|---|---|
| Steatosis | 0 | 1 |
| Inflammation | 0 | 1 |
| Fibrosis | 0 | 1 |
| Balloning | 0 | 0 |
| Necrosis | - | - |
| **Total score** | **0** | **3** |

**Steatosis**: hepatocytes exhibiting macro/microvacuolization, 0 (<5%); 1 (5-33%); 2 (33-66%); 4 (>66%). **Inflammation**: foci per 200x field, 0 (no foci); 1 (<2 foci per field); 2 (2-4 foci per field); 3 (>4 foci per field). **Fibrosis**: 0 (none); 1 (mild); 2 (moderate); 3 (severe); 4 (cirrhosis). **Ballooning**: 0 (none); 1 (few balloon cells); 2 (many balloon cells). **Necrosis**: not reported in NAFLD.

**Table S2.** Primer sequences used for Reverse Transcription Quantitative-PCR (RT-qPCR).

| **Gene name** | **Forward (5' - 3')** | **Reverse (5' - 3')** |
|---|---|---|
| *Acaca* | TTCCACGTAGGAGGAGCTTCC | CCTCCGGTGCCTTCTCATTAC |
| *Acly* | CTGTGCCACCATGTTCTCCTC | AGGCCTGGTTCTTGGCTACTG |
| *Atf4* | TTGTCCGTTACAGCAACACTG | GCAGCAGCACCAGGCTCT |
| *Bsep* | AAGGACAGCCACACCAACTC | CCAGAACATGACAAACGGAA |
| *Ccne1* | TTGCAAGACCCAGATGAAGA | TCCACGCATGCTGAATTATC |
| *Cldn1* | ATCACCTTCGGGAGCTCAGGT | TGATGGGGGTCAAGGGGTCAT |
| *Cldn2* | ATGCCTTCTTGAGCCTGCTT | CAGTGTCTCTGGCAAGCTGA |
| *Cldn3* | CGTACCGTCACCACTACCAG | CAGCCTAGCAAGCAGACTGT |
| *Cldn4* | CCATGGAACCCTTCCGTTGA | ACCCGTCCATCCACTCTACA |
| *Cldn6* | CCCTTGGTGGCTGATGCTCAA | AGGTGGAGCTTGGACTCAGGT |
| *Cldn7* | TGTACCTACCTGGTCCTGGG | TCTCAGAAAAGACGGGACGC |
| *Cldn8* | GGAATGCCAATCCATCACGC | CTCTTTTATCCCCAGGCCCC |
| *Cldn9* | AAGTGGTATGGGAGGGGCTGT | CGCAGGTGGAAGCTTCTGGAA |
| *Cldn12* | TGTGTGCAGATGTGCTCCTGT | GCAGGAGGGCTTGAGCTGTAT |
| *Cldn14* | CTAACCAGAGGGCATGTGTGC | AGTCCCATCCACCTTGATGCT |
| *Cldn15* | GGTGGCTATCTCGTGGTACG | GCACTCCAGCCCAAGTAGAG |
| *Cldn19* | GGAATTCTTCAACCCCCAGCAC | ATAGGGCTGTGGGATGCTGTT |
| *Chop* | CCACCACACCTGAAAGCAGAA | AGGTGAAAGGCAGGGACTCA |
| *Ctgf* | CAGACTGGAGAAGCAGAGCC | GCTTGGCGATTTTAGGTGTC |
| *Cyclophiline* | CAGACGCCACTGTCGCTT | TGTCTTTGGAACTTTGTCTGC |
| *Cyp4a14* | ACCCCTCTAGATTTGCACCA | AGCAAACTGTTTCCCAATGC |
| *Cyp4a10* | CACACCCTGATCACCAACAG | TCCTTGATGCACATTGTGGT |
| *Edem1* | CTACCTGCGAAGAGGCCG | GTTCATGAGCTGCCCACTGA |
| *Ehhadh* | CTATGATCCGCCTCTGCAA | TGGCTCTAACCGTATGGTCC |
| *Fabp1* | TGCAGAGCCAGGAGAACTTT | GATTTCTGACACCCCCTTGA |
| *Fgf21* | CTCCAGCAGTTCTCTGA | CCTGGGTGTCAAAGCCTCTA |
| *Fasn* | AGAAGAGCCATGGAGGAGGTG | ATGTCCACACCACCAATGAGG |
| *Grp78* | TTCAGCCAATTATCAGCAAACTCT | TTTTCTGATGTATCCTCTTCACCAGT |
| *Mlxipl* | CGGATACGGACTTGGAGGATC | GAAGTGTCCGCTGTGGATGAC |
| *Mt2* | TTGCGCTCGACCCAATACTC | CATTGTTTGCATTTGCAGGCG |
| *Ocln* | TCCGGATCCTGTCTATGCTCA | ATAGCCACCTCCGTAGCCAAA |
| *Perk* | TCATCCAGCCTTAGCAAACC | ATGCTTTCACGGTCTTGGTC |

| | | |
|---|---|---|
| *Pparcg1a* | AAGTGGTGTAGCGACCAATCG | AATGAGGGCAATCCGTCTTCA |
| *Reg3b* | CCCAAGGGCTCCCAGGCTTA | GAGGTGTCCTCCAGGCCTCT |
| *Reg3g* | TGGCGCTGAAGCTTCCTTCC | TCATAGCCCAGTGTCGGGTCA |
| *Retnlb* | CCATTTCCTGAGCTTTCTGG | AGCACATCCAGTGACAACCA |
| *Scd1* | GCTCTACACCTGCCTCTTCG | CAGCCGAGCCTTGTAAGTTC |
| *Shp* | CACGATCCTCTTCAACCCAG | AGACTTCACACAGTGCCCA |
| *Srebf1* | GGCTCTGGAACAGACACTGG | TGGTTGTTGATGAGCTGGAG |
| *Sult1e1* | AAACTCACCTGCCACCCAAG | TTGGCGTTCCGGCAAAGATA |
| *Xbp1s* | CTGAGTCCGAATCAGGTGCAG | GTCCATGGGAAGATGTTCTGG |
| *Tjp1* | GAAACTCTGCTGAGCCCCCTA | GTTTTAGGGTCACCCGACGAG |
| *Tjp2* | CGAAGCAGTCTGGGTCTCTGA | CCGGCTCCTCTAGCTCATTGT |
| *Tjp3* | ATGGTATGCCATTTCGGAACC | CCGGGTACAACGTGTCCACTA |
