# Supplementary material for: Lactational delivery of Triclosan promotes non-alcoholic fatty liver disease in newborn mice
Source: Nat Commun. 2022 Jul 27;13:4346. doi: 10.1038/s41467-022-31947-4 (PMC9329322; doi:10.1038/s41467-022-31947-4)
Supplement: Supplementary file 3 — Description of Additional Supplementary Files [file 41467_2022_31947_MOESM3_ESM.pdf]

Description of Additional Supplementary Files

File Name: Supplementary Data 1
Description: For each figure and supplementary figure, exact P values for all comparisons and group size for each group are listed.
