# Supplementary material for: Lactational delivery of Triclosan promotes non-alcoholic fatty liver disease in newborn mice
Source: Nat Commun. 2022 Jul 27;13:4346. doi: 10.1038/s41467-022-31947-4 (PMC9329322; doi:10.1038/s41467-022-31947-4)
Supplement: Supplementary file 5 — Reporting Summary [file 41467_2022_31947_MOESM5_ESM.pdf]

nature portfolio

Corresponding author(s): Robert Tukey

Last updated by author(s): Jun 24, 2022

Reporting Summary

Nature Portfolio wishes to improve the reproducibility of the work that we publish. This form provides structure for consistency and transparency in reporting. For further information on Nature Portfolio policies, see our Editorial Policies and the Editorial Policy Checklist.

Statistics

For all statistical analyses, confirm that the following items are present in the figure legend, table legend, main text, or Methods section.

n/a	Confirmed	
☐	☒	The exact sample size (*n*) for each experimental group/condition, given as a discrete number and unit of measurement
☐	☒	A statement on whether measurements were taken from distinct samples or whether the same sample was measured repeatedly
☐	☒	The statistical test(s) used AND whether they are one- or two-sided<br>*Only common tests should be described solely by name; describe more complex techniques in the Methods section.*
☒	☐	A description of all covariates tested
☐	☒	A description of any assumptions or corrections, such as tests of normality and adjustment for multiple comparisons
☐	☒	A full description of the statistical parameters including central tendency (e.g. means) or other basic estimates (e.g. regression coefficient) AND variation (e.g. standard deviation) or associated estimates of uncertainty (e.g. confidence intervals)
☐	☒	For null hypothesis testing, the test statistic (e.g. F, t, r) with confidence intervals, effect sizes, degrees of freedom and P value noted<br>*Give P values as exact values whenever suitable.*
☒	☐	For Bayesian analysis, information on the choice of priors and Markov chain Monte Carlo settings
☒	☐	For hierarchical and complex designs, identification of the appropriate level for tests and full reporting of outcomes
☒	☐	Estimates of effect sizes (e.g. Cohen's *d*, Pearson's *r*), indicating how they were calculated

Our web collection on statistics for biologists contains articles on many of the points above.

Software and code

Policy information about availability of computer code

Data collection	Liver histological pictures were taken in upright Zeiss microscope; Fluorescence liver images were taken in Leica Confocal Microscope. RNA-seq was performed in Illumina NovaSeq 6000 sequencer.
Data analysis	Staining:<br>Image J 2.3.0<br>Statistical analyzes:<br>GraphPad 9.1.0

For manuscripts utilizing custom algorithms or software that are central to the research but not yet described in published literature, software must be made available to editors and reviewers. We strongly encourage code deposition in a community repository (e.g. GitHub). See the Nature Portfolio guidelines for submitting code & software for further information.

Data

Policy information about availability of data

All manuscripts must include a data availability statement. This statement should provide the following information, where applicable:

- Accession codes, unique identifiers, or web links for publicly available datasets
- A description of any restrictions on data availability
- For clinical datasets or third party data, please ensure that the statement adheres to our policy

RNA-Seq data is deposit in NCBI Gene expression Omnibus (GEO) database under acession number GSE200705.

Field-specific reporting

Please select the one below that is the best fit for your research. If you are not sure, read the appropriate sections before making your selection.

☒ Life sciences ☐ Behavioural & social sciences ☐ Ecological, evolutionary & environmental sciences

For a reference copy of the document with all sections, see nature.com/documents/nr-reporting-summary-flat.pdf

Life sciences study design

All studies must disclose on these points even when the disclosure is negative.

Sample size	No power analyzes were used to predetermine samples sizes. Samples sizes were chosen based on prior literature using similar experiments. (Nature Communications. 2020, PMID: 32839596)
Data exclusions	We excluded PERK inhibitor (GSK2606414) in vivo experiment, because the dosage used was low and had no effect on the PERK-ATF4 pathway.
Replication	Two technical replicates or more (from different litters and dates), as well as biological replicates were performed to ensure data reproducibility. All replication were successful.
Randomization	All samples were used for Q-RT-PCR and we chose random samples for Western Blotting to make sure Q-RT-PCR and protein analysis have the same result.
Blinding	We had no specific methods to blind the investigators during the experiments, but all mice were treated equally at the same time.

Reporting for specific materials, systems and methods

We require information from authors about some types of materials, experimental systems and methods used in many studies. Here, indicate whether each material, system or method listed is relevant to your study. If you are not sure if a list item applies to your research, read the appropriate section before selecting a response.

Materials & experimental systems

n/a	Involved in the study	
☐	☒	Antibodies
☒	☐	Eukaryotic cell lines
☒	☐	Palaeontology and archaeology
☐	☒	Animals and other organisms
☒	☐	Human research participants
☒	☐	Clinical data
☒	☐	Dual use research of concern

Methods

n/a	Involved in the study	
☒	☐	ChIP-seq
☒	☐	Flow cytometry
☒	☐	MRI-based neuroimaging

Antibodies

Antibodies used	Antibodies used for Western Blotting were: GAPDH (Santa Cruz, sc-32233), β-catenin (Santa Cruz, sc-7963), claudin-1 (Santa Cruz, sc-166338), AMPKα1/2 (Santa Cruz, sc-25792), CYP7A1 (Abcam, ab-65596), SCD1 (Santa Cruz, sc-14720), phospho-AMPK (Cell Signaling, CS2535), phospho-eIF2α (Cell Signaling, CS3597), ATF4 (Cell Signaling, CS11815), IL-1β (Cell Signaling, CS12426), cleaved caspase 3 (Cell Signaling, CS9661), FASN (Cell Signaling, CS3180), FOXO1 (Cell Signaling, CS2880), NQO-1 (Cell Signaling, CS62262), cytochrome C (Cell Signaling, CS11940), caspase-9 (Cell Signaling, CS9508S), phospho-β-Catenin (Cell Signaling, CS5651), LGR5 (Abclonal, A10545), SGK1 (Abclonal, A3936). The secondary antibodies anti-mice IgG horseradish peroxidase (HRP) conjugated antibody and anti-rabbit IgG HRP conjugated antibodies were obtained from Cell Signaling Technology, Inc. (Danvers, MA). All primary antibodies were diluted 1:1,000 and secondary antibodies were diluted 1:3,000.<br>Antibodies used for immunohistochemistry were: Ki-67 (GeneTex, GTX16667) and F4/80 (ThermoFisher, 41-4801-82). The secondary antibodies Biotinylated antibodies anti-rabbit and anti-mouse were obtained by Pharmingen. For IHC the primary antibodies were dilluted 1:100 and secondary 1:200.
Validation	Exact sources and catalog number are provided. We confirmed that all antibodies reacted with a single protein and expected molecular weight. All antibodies used were published in other studies validating its use.

Animals and other organisms

Policy information about studies involving animals; ARRIVE guidelines recommended for reporting animal research

Laboratory animals	Male C57BL/6 (Age: 21 days) (strain: Wild-type, Atf4-/-, Pparα-/-, gp130Act). All mice were housed in a specific pathogen–free facility

March 2021

Laboratory animals	with a 12-h light, 12-h dark cycle and given free access to food and water
Wild animals	No wild animals were used.
Field-collected samples	No field collected samples were used.
Ethics oversight	The protocols for mice handling and procedures were approved by the UCSD Animal Care and Use Committee (IACUC), and these protocols were conducted in accordance with federal regulations. Animal Protocol S99100 was approved by the UCSD Institutional Animal Care and Use Committee.

Note that full information on the approval of the study protocol must also be provided in the manuscript.
